# Simultaneous surgical resection of cardiac myxoma and atypical thymic carcinoid: a case report

**DOI:** 10.1186/s13256-021-03091-y

**Published:** 2021-10-06

**Authors:** Ryohei Matsushima, Kosuke Fujino, Eri Matsubara, Yoshiko Masuda, Koei Ikeda, Makoto Suzuki, Toshihiro Fukui

**Affiliations:** 1 Department of Thoracic Surgery, Kumamoto University Hospital, 1-1-1 Honjo, Chuo-ku, Kumamoto, Japan; 2 Cardiovascular Surgery, Kumamoto University Hospital, 1-1-1 Honjo, Chuo-ku, Kumamoto, Japan

**Keywords:** Cardiac myxoma, Thymic carcinoid, Surgery

## Abstract

**Background:**

Cardiac myxoma is the most common type of primary cardiac tumor, and thymic carcinoid is a rare neuroendocrine tumor. No previous reports have described surgical management of concomitant occurrence of these neoplasms. We report a case of simultaneous surgical resection in a patient with coexisting cardiac myxoma and atypical thymic carcinoid.

**Case presentation:**

A 44-year-old Japanese woman underwent chest roentgenography revealing an abnormality in the mediastinum. Computed tomography revealed a 100 mm mass in the anterior mediastinum and also a 30 mm mass in the left atrium. The mediastinal tumor was diagnosed as atypical carcinoid by biopsy. Having completed resection of atypical thymic carcinoid, cardiac mass was successfully resected with careful consideration of minimizing operation time and optimizing patient safety and oncological treatment. The histopathological diagnosis of the cardiac mass was myxoma. No adjuvant chemotherapy was administered, and no recurrence was seen as of the 45 month follow-up.

**Conclusions:**

The simultaneous surgery of cardiac myxoma and atypical thymic carcinoid was feasible and effective. To the best of our knowledge, this is the first case report to describe one-stage treatment of these neoplasms.

## Background

Anterior mediastinal tumors are often found incidentally during scrutiny of other diseases, but few cases have described surgical treatment of concomitant cardiac and mediastinal tumors. Cardiac myxoma is the most common type of primary cardiac tumor in adults, and warrants complete surgical resection owing to the risks of sudden death and embolism [1]. Thymic carcinoid accounts for 2% of all mediastinal tumors, and is rarer and more malignant than pulmonary carcinoid [2, 3]. Patients with atypical thymic carcinoid, which is usually categorized as an intermediate-grade malignancy, show a more aggressive clinical course than patients with typical carcinoid [4]. The standard treatment of thymic carcinoid in the absence of distant metastasis involves definitive oncological resection [2–5]. We report herein a case of simultaneous resection of cardiac myxoma and atypical thymic carcinoid via mediastinal sternal incision.

## Case presentation

A 44-year-old Japanese woman underwent chest roentgenography during a regular health checkup, revealing an abnormality in the left mediastinum. The patient had a medical history of hypertension and uterine myoma, and she was taking angiotensin II receptor blocker and calcium channel blocker. Her family had no medical history. She was married with gravida 2, para 2. She did not consume alcohol or tobacco. The initial vital signs were a temperature 36.5 °C, blood pressure 130/91 mmHg, heart rate 84 beats per minute, respiratory rate 17 breaths per minute, and saturation 99% on room air. No significant findings were obtained from physical and neurological examination. Her initial blood work revealed hemoglobin of 146 g/L, leukocytes of 6.8 × 10^3^/μL, platelets of 348 × 10^3^/μL, creatinine of 0.59 mg/dL, aspartate aminotransferase of 26 U/L, alanine aminotransferase of 38 U/L, lactate dehydrogenase of 123 U/L, soluble interleukin-2 receptors of 177 U/mL, carcinoembryonic antigen of 2.0 ng/mL, cytokeratin fragment (CYFRA) of 1.3 ng/mL, and B-type natriuretic peptide of 6.5 pg/mL.

**Fig. 1:**
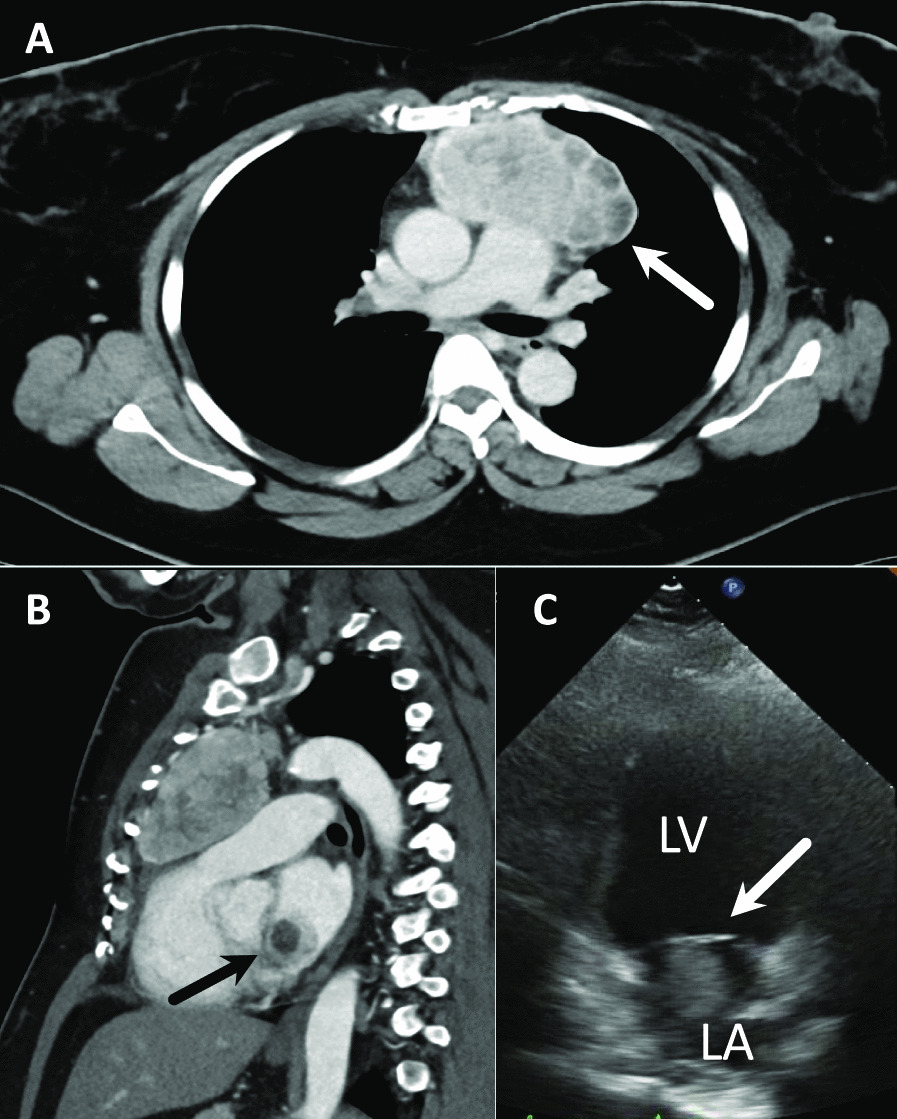
**A** Chest computed tomography showing a 100 × 90 mm anterior mediastinal mass with heterogeneous contrast (arrows). **B** Sagittal-view computed tomography. A 30 × 30-mm mass is identified in the left atrium (arrows). **C** Transthoracic echocardiography showing a mobile mass on the mitral valve. *LV* left ventricular, *LA* left atrium

Subsequent computed tomography (CT) of the chest revealed a 100 × 90 mm mass showing heterogeneous contrast enhancement in the anterior mediastinum, with no invasion into surrounding organs (Fig. 1A). A 30 × 30 mm mass was also shown in the left atrium (Fig. 1B). No lymph node metastases or distant metastases were identified. Transthoracic echocardiography showed a movable mass attached to the mitral valve, prolapsing into the left ventricle (Fig. 1C). CT-guided needle biopsy (CTNB) diagnosed the mediastinal mass as atypical thymic carcinoid.

**Fig. 2:**
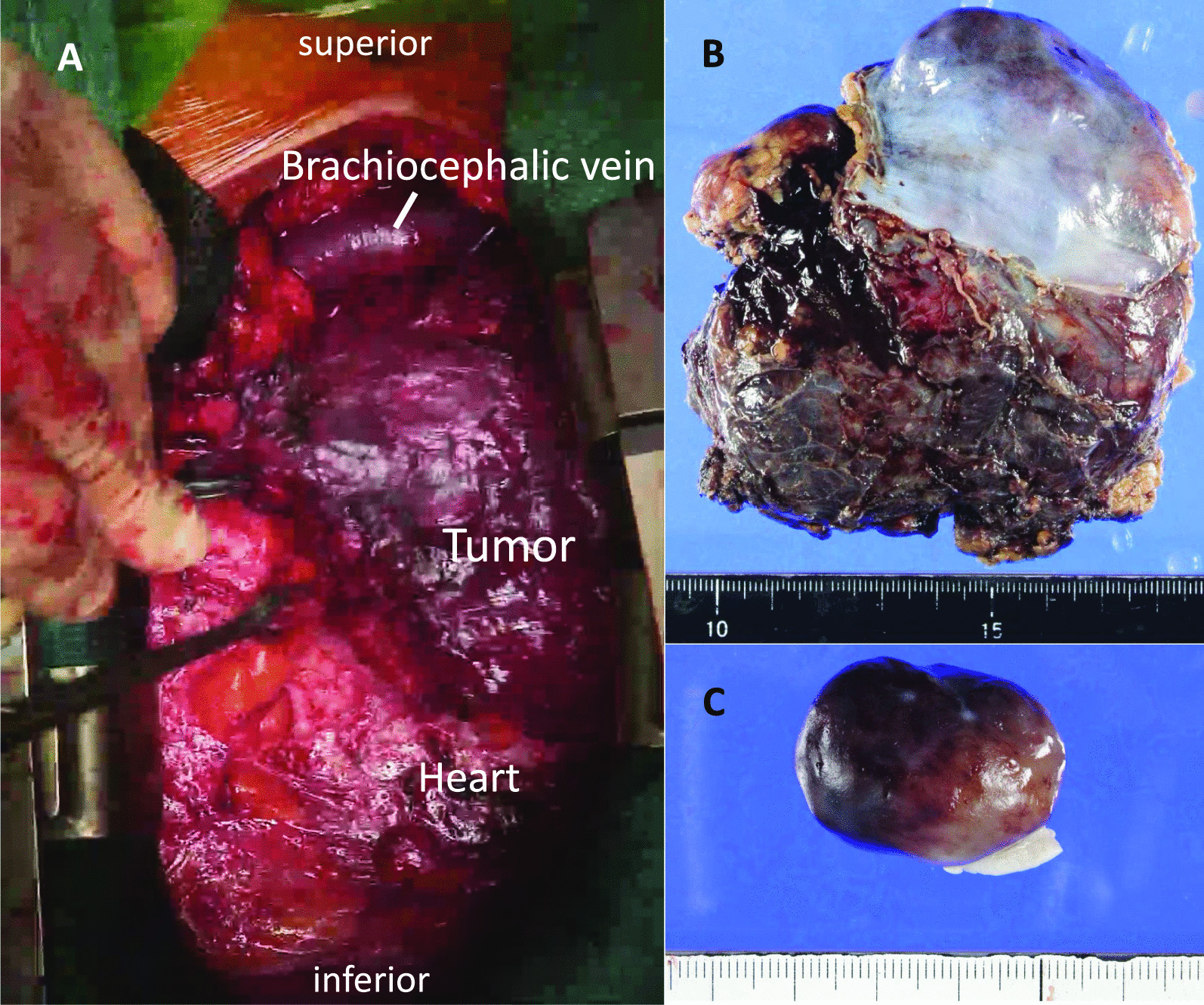
**A** Intraoperative view showing the mediastinal tumor in front of the heart and ascending aorta. **B** Macroscopic view of the mediastinal tumor. **C** Macroscopic view of the intracardiac tumor

**Fig. 3:**
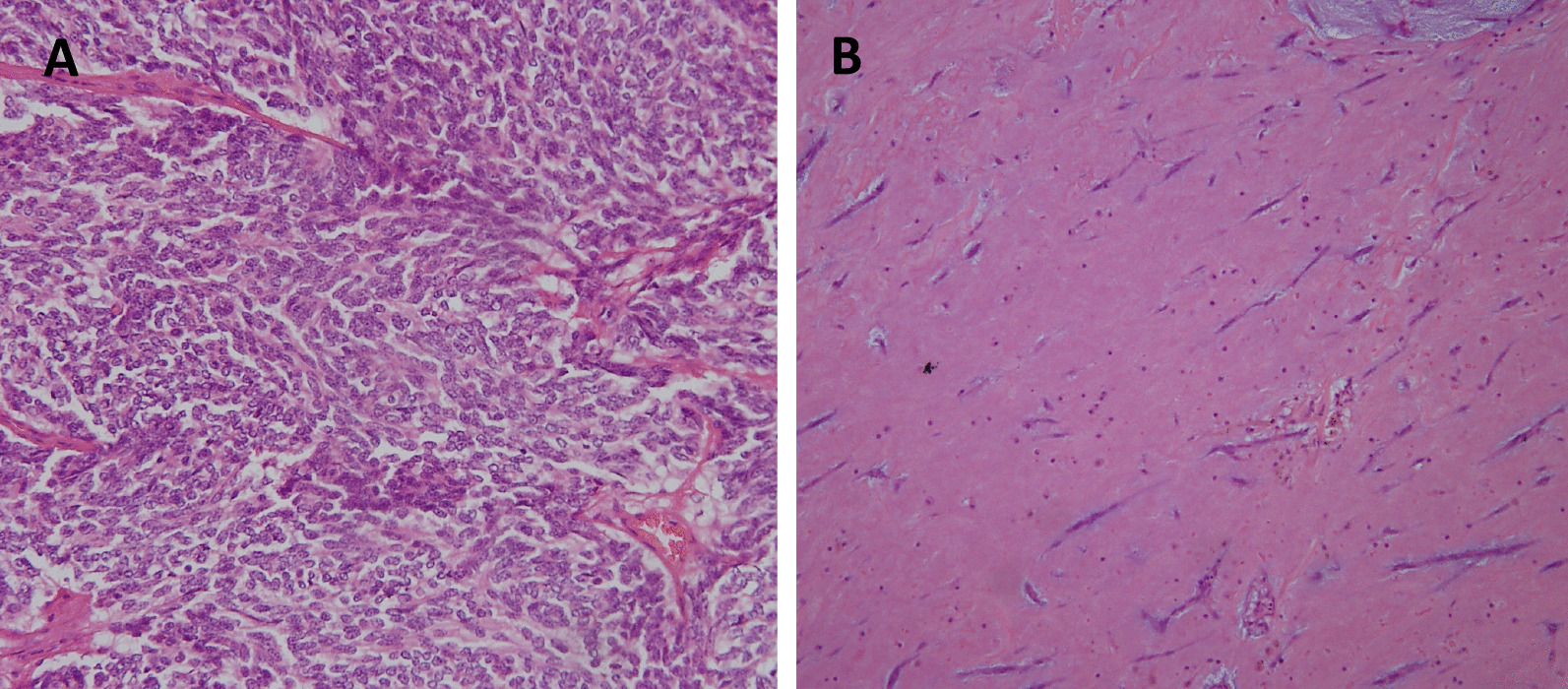
**A** Hematoxylin and eosin staining of the mediastinal tumor. Spindle-shaped tumor cells with small and round nuclei demonstrate solid and trabecular growth patterns, indicating thymic neuroendocrine carcinoid. **B** Hematoxylin and eosin staining of the cardiac tumor. Scattered spindle cells against a background of loose myxoid stroma indicate cardiac myxoma

The patient underwent simultaneous resection of the cardiac myxoma and atypical thymic carcinoid via a midline sternotomy. The mediastinal tumor was located on the left lobe of the thymus, but showed no invasion into surrounding organs (Fig. 2A). After complete resection without damaging the mediastinal tumor (Fig. 2B), we cleaned the surgical field with physiological saline and changed the surgical devices to prevent any inadvertent scattering of microscopic tumor cells. The left atrial pedunculated tumor had developed from the interatrial septal oval fossa. The cardiac tumor was successfully resected, and atrial structures remained intact (Fig. 2C). The time of cardiopulmonary bypass, aortic clamp, and surgery was 68, 36, and 270 minutes, respectively. Histopathological examination of the resected tumor revealed atypical thymic carcinoid and cardiac myxoma (Fig. 3A, B). The patient recovered well and was discharged 13 days postoperatively. No adjuvant chemotherapy was administered. The patient was followed up regularly with physical examination, chest roentgenography, echocardiography, and CT scan every 6 months, and no recurrence was seen as of the 45 month follow-up.

## Discussion

Although clinicians sometimes encounter patients with concomitant cardiac disease and other malignancies, the clinical approach should be decided based on the individual case because definitive treatment has not been established. There were no other reports that described simultaneous surgical resection of cardiac myxoma and thymic carcinoid in PubMed with the keywords “myxoma” and “carcinoid.” We report a case of complete resection of these tumors via mediastinal sternal incision with good clinical outcome.

About 75% of cardiac tumors are benign, and cardiac myxomas occupy about half of the cardiac benign tumors [6]. However, cardiac myxomas are life-threatening tumors owing to the risk of embolization and sudden death, and should be resected prior to treatment of other tumors [1]. Atypical thymic carcinoids are considered to be intermediated-grade malignant tumors and have poor prognosis. Jia *et al.* reported that the median overall survival of atypical thymic carcinoid was 3.6–6.4 years [2]. Although the rarity of thymic carcinoids has resulted in a lack of extensive data, radical surgery is regarded as the only curative method. Patients whose tumor could not be completely removed had a shorter overall survival than those who underwent R0 resection [7]. There are currently no data to support neoadjuvant chemoradiotherapy for resectable thymic carcinoids.

In the present case, we were able to diagnose both cardiac myxoma and atypical thymic carcinoid from the result of transthoracic echocardiography and CTNB before treatment. Approximately 100 mm of mediastinal tumor was localized in front of the heart, preventing open-heart surgery. We therefore performed simultaneous resection immediately without neoadjuvant therapy. The risk of systematic spread of the atypical carcinoid cell during the one-stage surgery had to be considered, since extracorporeal circulation (ECC) was needed during cardiac tumor resection. A two-stage surgery may have offered the advantage of a lower risk of scattering tumor cells, but would also have disadvantages such as a longer total duration of therapy and potential difficulties in the second stage due to adhesions. Several groups have reported surgical approaches for combined heart diseases and other malignancies, and concluded that simultaneous surgery was not associated with any adverse impacts on morbidity, mortality, or recurrence-free survival [5, 8]. On the other hand, Hasegawa *et al.* reported a case involving suspected hematogenous dissemination of malignant tumor cells via ECC [9]. They pointed out that using aspirated blood for ECC could increase the risk of tumor cell dissemination. As a strategy to prevent tumor cell scattering, we initially performed en bloc resection of the thymus along with the mediastinal tumor, then cleaned the surgical field sufficiently with physiological saline and changed all surgical devices before performing cardiac surgery. Further, we reduced the use of aspirated blood as much as possible during ECC.

As this is the first report of coexisting cardiac myxoma and atypical thymic carcinoid, the relationship between these tumors is unclear. Approximately 7% of cardiac myxomas arise in patients with Carney complex (CNC), an inherited disorder characterized by spotty skin pigmentation, endocrine overactivity, and myxoma [10]. Various endocrine tumors, including thyroid tumors, testes tumor, and ovary tumors, are also known to arise in patients with CNC. Atypical thymic carcinoids are a rare type of neuroendocrine tumor, and CNC may thus have been a possibility in this patient. However, no other significant clinical findings were suggestive of CNC, such as hormonal abnormalities or family history, and the patient declined genetic examination.

Additional postoperative therapy is not necessarily needed as long as the tumors were removed completely, though we believe that postoperative chemotherapy for thymic carcinoid that could not be removed completely and postoperative radiation therapy in the case of capsular invasion, positive margins, or positive lymph nodes are efficacious [11, 12]. In the present case, we did not give any postoperative therapies, and no findings of recurrence were detected as of the 45 month follow-up. CNC is reportedly associated with a high rate of recurrence for cardiac myxomas [13]. Although the relationship with CNC in the present case remains uncertain, we will continue careful observation for not only the thymic carcinoid but also the cardiac myxoma.

## Conclusions

We encountered a case in which simultaneous surgery enabled complete oncological resection without any recurrence as of 45 months postoperatively. Because cases of concomitant cardiac myxoma and thymic carcinoid are extremely rare, definitive treatment has yet to be established. We will continue follow-up for the long term, but further case accumulation is needed.

## Data Availability

All the data are available within the article.

## Abbreviations

CT: Computed tomography
CTNB: Computed tomography-guided needle biopsy
ECC: Extracorporeal circulation
CNC: Carney complex
